# Hierarchical Modeling of the Local Reaction Environment in Electrocatalysis

**DOI:** 10.1021/acs.accounts.4c00234

**Published:** 2024-07-20

**Authors:** Xinwei Zhu, Jun Huang, Michael Eikerling

**Affiliations:** † Theory and Computation of Energy Materials (IEK-13), Institute of Energy and Climate Research, Forschungszentrum Jülich GmbH, 52425 Jülich, Germany; ‡ Chair of Theory and Computation of Energy Materials, Faculty of Georesources and Materials Engineering, RWTH Aachen University, 52062 Aachen, Germany; § Theory of Electrocatalytic Interfaces, Faculty of Georesources and Materials Engineering, RWTH Aachen University, 52062 Aachen, Germany

## Abstract

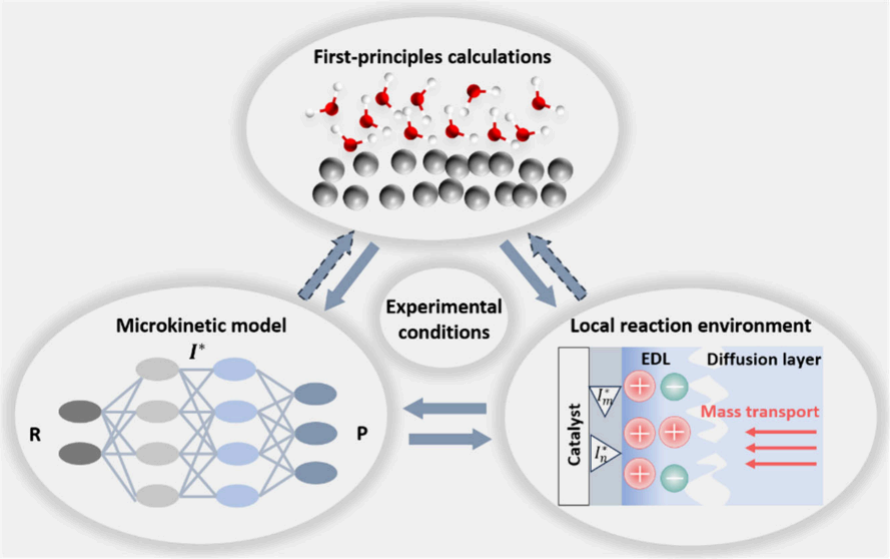

Electrocatalytic reactions, such as oxygen reduction/evolution reactions and CO_2_ reduction reaction that are pivotal for the energy transition, are multistep processes that occur in a nanoscale electric double layer (EDL) at a solid–liquid interface. Conventional analyses based on the Sabatier principle, using binding energies or effective electronic structure properties such as the d-band center as descriptors, are able to grasp overall trends in catalytic activity in specific groups of catalysts. However, thermodynamic approaches often fail to account for electrolyte effects that arise in the EDL, including pH, cation, and anion effects. These effects exert strong impacts on electrocatalytic reactions. There is growing consensus that the local reaction environment (LRE) prevailing in the EDL is the key to deciphering these complex and hitherto perplexing electrolyte effects. Increasing attention is thus paid to designing electrolyte properties, positioning the LRE at center stage. To this end, unraveling the LRE is becoming essential for designing electrocatalysts with specifically tailored properties, which could enable much needed breakthroughs in electrochemical energy science.

Theory and modeling are getting more and more important and powerful in addressing this multifaceted problem that involves physical phenomena at different scales and interacting in a multidimensional parametric space. Theoretical models developed for this purpose should treat intrinsic multistep kinetics of electrocatalytic reactions, EDL effects from subnm scale to the scale of 10 nm, and mass transport phenomena bridging scales from <0.1 to 100 μm. Given the diverse physical phenomena and scales involved, it is evident that the challenge at hand surpasses the capabilities of any single theoretical or computational approach.

In this Account, we present a hierarchical theoretical framework to address the above challenge. It seamlessly integrates several modules: (i) microkinetic modeling that accounts for various reaction pathways; (ii) an LRE model that describes the interfacial region extending from the nanometric EDL continuously to the solution bulk; (iii) first-principles calculations that provide parameters, *e.g*., adsorption energies, activation barriers and EDL parameters. The microkinetic model considers all elementary steps without designating an *a priori* rate-determining step. The kinetics of these elementary steps are expressed in terms of local concentrations, potential and electric field that are codetermined by EDL charging and mass transport in the LRE model. Vital insights on electrode kinetic phenomena, *i.e*., potential-dependent Tafel slopes, cation effects, and pH effects, obtained from this hierarchical framework are then reviewed. Finally, an outlook on further improvement of the model framework is presented, in view of recent developments in first-principles based simulation of electrocatalysis, observations of dynamic reconstruction of catalysts, and machine-learning assisted computational simulations.

## Key References

- HuangJ.; ZhuX.; EikerlingM.The Rate-Determining Term of Electrocatalytic Reactions with First-Order Kinetics. Electrochim. Acta2021, 393, 13901910.1016/j.electacta.2021.139019.^1^*Analytical expressions of the activity of multistep reactions with first-order kinetics were derived under steady-state conditions*. *Expressions for inverse rates allow the rate-determining term (RDT) to be identified, a concept that is insightful in analyzing Tafel slopes and volcano plots*.
- HuangJ.; LiM.; EslamibidgoliM. J.; EikerlingM.; GroßA.Cation Overcrowding Effect on the Oxygen Evolution Reaction. JACS Au2021, 1, 175210.1021/jacsau.1c0031534723278 PMC8549051.^2^*Application of the hierarchical framework to the oxygen evolution reaction with a focus on cation effects*. *The observed decrease in activity with increasing effective size of cations was interpreted as a consequence of cation overcrowding near negatively charged electrode surface*.
- ZhuX.; HuangJ.; EikerlingM.Electrochemical CO_2_ Reduction at Silver from a Local Perspective. ACS Catal.2021, 11( (23), ), 14521–1453210.1021/acscatal.1c04791.^3^*Application of the hierarchical framework to understand kinetic phenomena observed in electrochemical CO*_2_*reduction at silver, such as potential-dependent Tafel slopes, cation effects and bicarbonate effects, from the perspective of the local reaction environment*.
- ZhuX.; HuangJ.; EikerlingM.pH Effects in a Model Electrocatalytic Reaction Disentangled. JACS Au2023, 3( (4), ), 1052–106410.1021/jacsau.2c0066237124300 PMC10131201.^4^*Systematic comparison of the hierarchical framework and its simplified variants allows us to disentangle interwoven factors influencing pH effects in formic acid oxidation. The bell-shaped activity-pH relation in phosphate solution and the trapezoidal-shaped activity-pH relation in perchlorate solution, are deciphered*.

## Introduction

**Figure 1:**
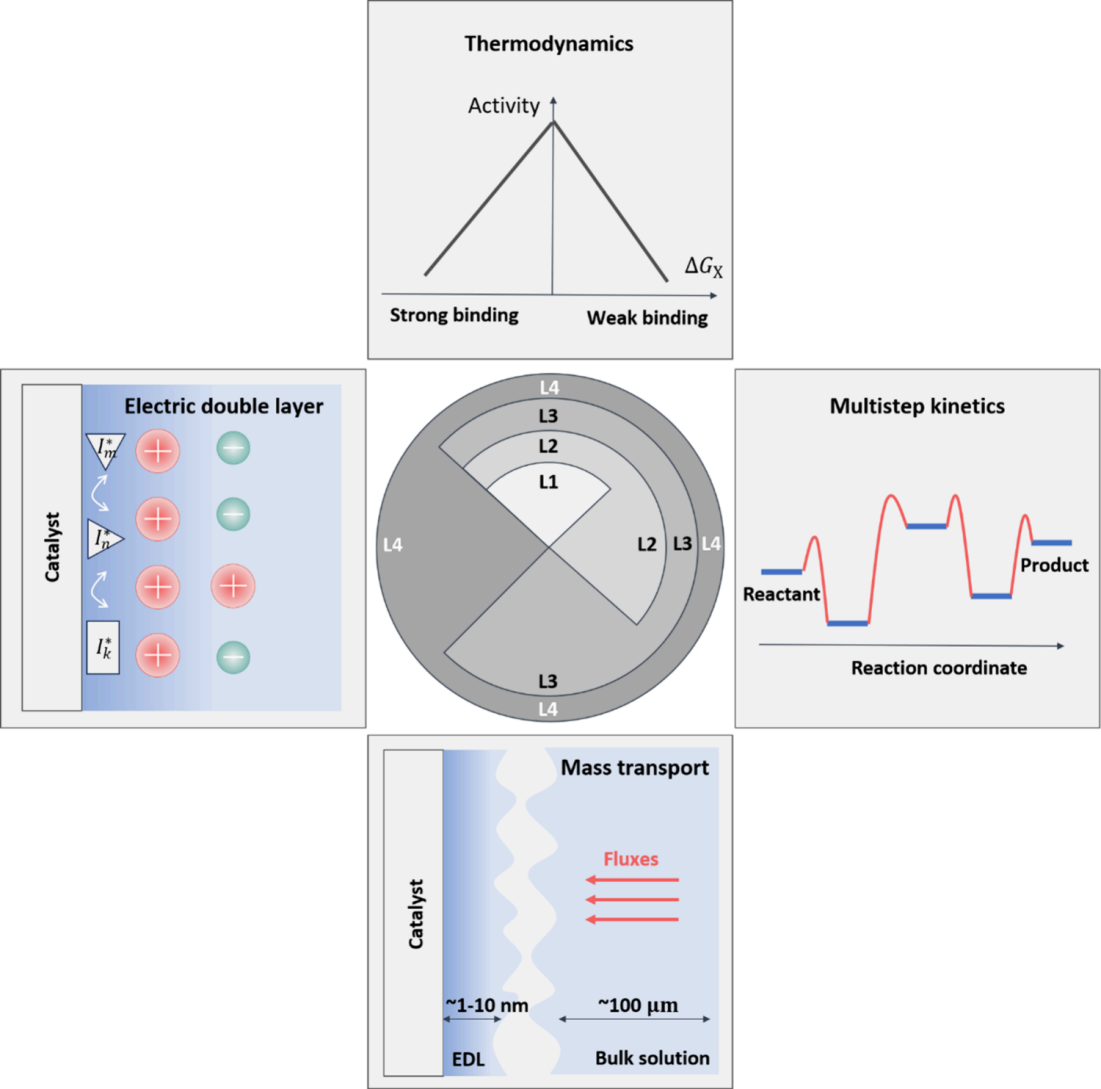
Four crucial components, *i.e*., thermodynamics of elementary steps, multistep kinetics, mass transport phenomena, and electric double layer, constitute a proper understanding of the activity and selectivity of electrocatalytic reactions. Existing theoretical methodologies for electrocatalytic reactions are categorized into four levels based on the components treated. Models on level 1 (L1) consider only thermodynamics, while those on level 2 (L2) incorporate both thermodynamics and kinetics of multiple steps into a microkinetic model. Level 3 (L3) improves over L1 and L2 further by integrating the macroscopic mass transport in the electrolyte solution into the microkinetic model. Finally, level 4 (L4) completes the circle by adding electric double layer effects.

Electrocatalysis stands as the cornerstone discipline to deliver breakthroughs in electrochemical energy conversion technologies, including fuel cells as well as carbon dioxide reduction, nitrate reduction and water splitting electrolyzers.^5^ Nevertheless, crucial electrocatalytic reactions grapple with sluggish kinetics and inadequate selectivity. A fundamental understanding of reaction mechanisms and factors that limit activity and selectivity toward targeted products is imperative in order to prompt progress in electrocatalyst design and development.^5^ However, these endeavors are complicated and hindered by the intricate multistep mechanisms and concurrent interrelated factors arising on multiple scales. Figure 1 depicts four essential components of a comprehensive understanding of electrocatalytic reactions:

- Multistep thermodynamics. The thermodynamics of an elementary step are determined by binding energies of adsorbed intermediates involved in this step. Past approaches have correlated the overall activity and selectivity of a specific reaction with the binding energies of key intermediates, which are readily calculated using first-principles based methods.^6−8^ This line of research leads to the development of tools for screening catalysts, employing for instance the d-band model^6^ or the generalized coordinate number model.^8^
- Multistep kinetics. Beyond thermodynamics, kinetic parameters, including but not limited to activation barriers, transfer coefficients and preexponential factors, are important for a quantitative understanding of electrocatalysis.^9^ In a few recent reports, these kinetic factors have been shown to change the qualitative trend of activity.^10−12^ For instance, in a microkinetic analysis accounting for activation barriers and transfer coefficients of elementary steps, the peak of the volcano plot on the binding energy axis changes with electrode potential.^10,11^ In the quest to simplify microkinetic analyses, it has become a customary practice to identify a single step that governs the overall rate of the reaction, termed the rate-determining step (RDS).^13,14^ The transition of the RDS from one step to another is often regarded as the cause of potential-dependent Tafel slopes.^13^
- Electric double layer effects. Electrocatalytic reactions take place in the electric double layer (EDL) at the catalyst-electrolyte interface.^15−19^ There exist many EDL effects, including the classical Frumkin corrections (*i.e*., the effects on potential and reactant concentration at the reaction plane),^15^ field-dependent adsorption energies of intermediates,^18,19^ and dependency of the solvent reorganization energy on the surface charge density.^20,21^ In addition to these equilibrium EDL effects, nonequilibrium EDL effects, first proposed by Levich et al. in the 1950s,^22^ are resurfacing in recent studies.^23^
- Mass transport. The consumption (production) of reactants (products) significantly influences local reactant/product concentrations and pH in the near-surface region.^4^ Recent progress has enabled direct probes of changes in ion concentrations and pH with a spatial resolution down to a few hundreds of nanometers.^24−26^

We categorize existing theoretical methodologies for modeling electrocatalytic reactions into four levels, as summarized in Figure 1. Level 1 (L1) considers only thermodynamics. Specifically, L1 models focus on the potential energy profiles of electrocatalytic reactions with the binding energies of intermediates that can be readily calculated from density functional theory (DFT)-based first-principles simulations.^5,7^ Despite the simplicity, L1 models can explain, surprisingly well, overall trends of activity and selectivity within groups of catalysts with similar electronic structures. The success of these models is evident from effective and easy-to-implement tools for screening catalyst materials, encompassing the d-band model,^6^ the generalized coordinate number,^8^ and volcano plots.^27^ In view of the simplicity of L1 models, it is unsurprising that counterexamples have been reported in the literature.^12^ For instance, the volcano plot predicts a sequence Pt(111) > Pt(100) > Pt(110) > Pt(211) for the oxygen reduction reaction (ORR), yet experiments show a trend Pt(211) > Pt(110) > Pt(111) > Pt(100).^12^ Additionally, concerns have been raised that the thermodynamics-based method may yield inaccurate results, due to the neglect of kinetic parameters of elementary steps, *e.g*., activation barriers and transfer coefficients.^11^

As an obvious step, kinetic factors are incorporated into L1 models on level 2 (L2). L2 models treat the kinetics on two sublevels. The first sublevel relies on the RDS concept, and only the kinetics of the RDS is considered.^13^ Practically, the RDS is often identified based on Tafel slope analysis that is, however, problematic. Values of Tafel slope can vary to a great extent among different measurements, leading to disparate reaction mechanisms reported in different studies.^13,28,29^ Furthermore, the Tafel slope exhibits high sensitivity to adsorbate coverages.^1,13,30^ Therefore, the RDS usually changes with electrode potential. These inconsistencies necessitate a full microkinetic model that treats the thermodynamics and kinetics of all elementary steps without singling out an RDS, constituting the second sublevel on the L2. These models have been utilized to rationalize potential-dependent Tafel slopes and volcano plots for various reactions.^1,11,31^

Recent years have witnessed a growing awareness of the significance of electrolyte composition. Various electrolyte effects, including cation,^32−34^ anion,^17,35^ and pH effects,^35−38^ have been observed for many reactions. Notably, most models on L1 and L2 fall short in interpreting the electrolyte effects, as they often ignore the role of the electrolyte. Consensus is growing that these electrolyte effects originate from the change of the local reaction environment (LRE) at the catalyst-electrolyte interface, which is shaped by the interplay of macroscopic mass transport and microscopic EDL charging. Therefore, resolving the LRE and its influence on the multistep kinetics has transpired as the focal point for further improvement, as emphasized in a recent Account of Xu et al.,^17^

“*The interplay of intrinsic microkinetics, homogeneous reactions, and mass transport limitations in determining the overall activity needs to be investigated in coupled transport–kinetic models*.”

Following the classical works on the modeling of catalyst layers in fuel cells,^39,40^ refined models on level 3 (L3) incorporating mass transport into L2 models have recently been applied to the CO_2_ reduction reaction (CO_2_RR),^41^ hydrogen evolution/oxidation reaction (HER/HOR),^42^ and oxygen evolution reaction (OER).^43^ L3 models yield the local pH and reactant concentration in the diffusion layer (0.1–100 μm), the scale for models to meet experimental measurements.^24−26^ For instance, Monteiro et al. measured the local pH during CO_2_RR at a distance of 80 μm from catalyst surface, and showed the consistency with numerical simulations.^25^

While L3 models are often claimed to be able to calculate local concentrations, it should be emphasized that the term “local” refers here to a macroscopic perspective with a relevant scale of ∼100 nm. There is thus a gap to the microscopic reacting zone that is located in the EDL. The EDL is not resolved in L3 models, while recent experimental evidence point to the central role of the EDL in understanding electrolyte effects.^44−46^ Incorporating EDL effects into L3 models to achieve a unified treatment of all components on level 4 (L4) has been attempted in recent works.^2−4,18,47^ In this Account, we introduce our approach to this L4 integration challenge. In the next section, we outline the framework of our approach, highlighting important know-how of handling the coupling between different module components. Afterward, the framework is employed to rationalize potential-dependent Tafel slopes, cation effects, and pH effects that are hot topics of current discussions. Applications will cover ORR, CO_2_RR, OER, formic acid oxidation reaction (FAOR), and hydrogen peroxide reduction reaction (HPRR). In the end, we share our perspective on how to further the integration of theory and computation in L4 models.

## Setting the Framework

**Figure 2:**
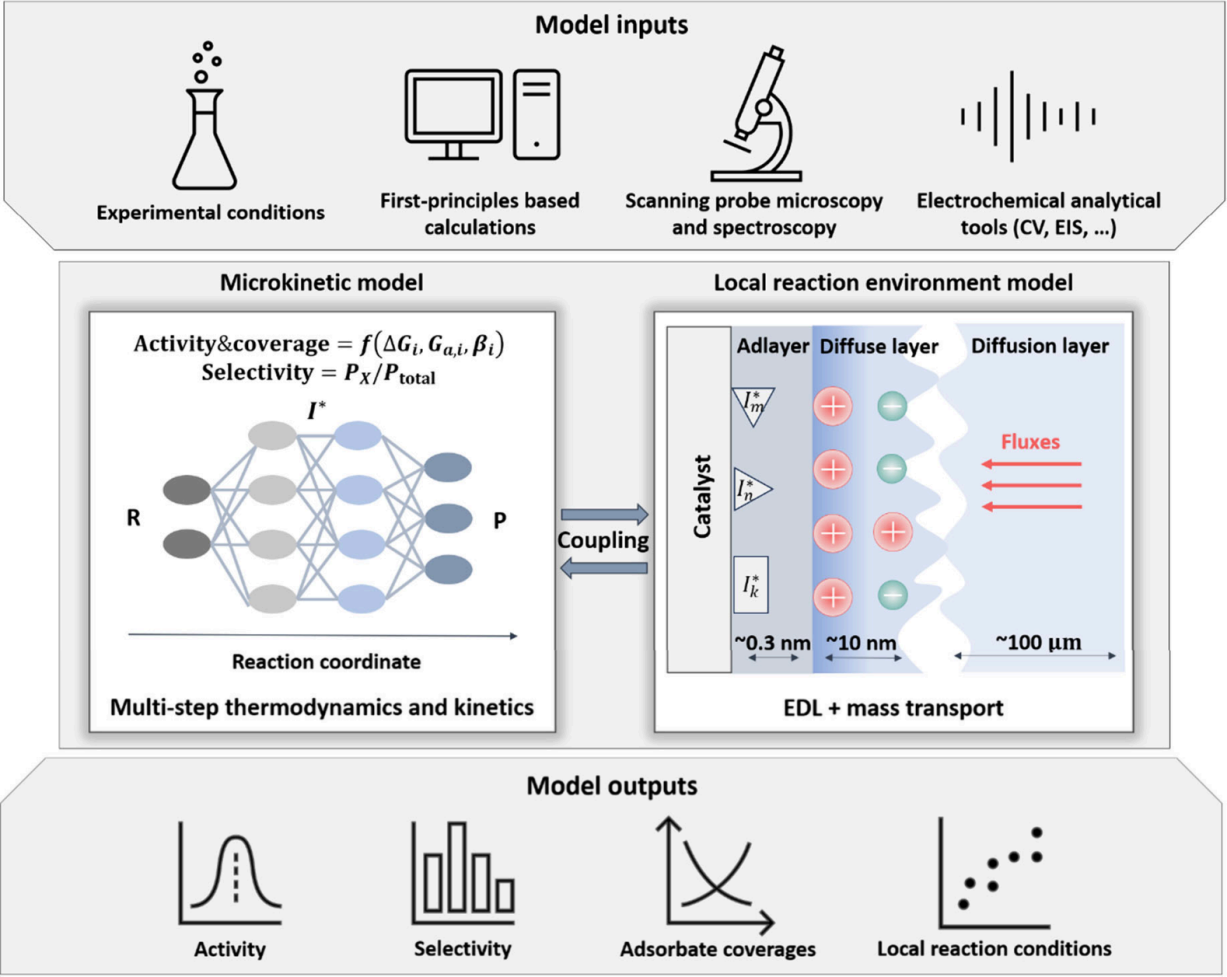
Hierarchical framework for modeling electrocatalytic reactions. The framework consists of two interrelated submodels, including a microkinetic model that accounts for the thermodynamics and kinetics of all elementary steps, and a submodel for the LRE that accounts for microscopic EDL effects and mass transport effects. These two submodels are connected through boundary conditions and are solved in a self-consistent manner, *e*.*g*., using COMSOL Multiphysics. The model inputs include the experimental conditions, the reaction mechanisms derived from first-principles based calculations, spectroscopic experiments and analytical tools, the energy parameters for reaction paths, *e.g*., adsorption energies and activation barriers, obtained from DFT calculations, and the EDL parameters extracted from AIMD simulations. The model outputs include activity, selectivity, adsorbate coverages, and local reaction conditions, including surface charging relation, reactant distribution, pH distribution, potential distribution, and electric field.

The framework comprises two essential submodels, as illustrated in Figure 2. The first one is a microkinetic model that integrates the thermodynamics and kinetics of all elementary steps. The second one processes the LRE, encompassing microscopic EDL effects and mass transport in solution. The two submodels are coupled via boundary conditions at the most probable reaction plane (RP) that is located in close proximity to the surface of the electrocatalyst. Typically, the outer Helmholtz plane is chosen as the RP for the convenience of applying Frumkin corrections.^19,48^ However, it has also been proposed that the position of the RP should be considered as a function of the overpotential.^49^

The microkinetic model requires *a priori* knowledge of the reaction mechanism that is usually inferred by combining key intermediates identified from spectroscopic experiments and first-principles-based calculations. A specific reaction mechanism is expressed as a series of elementary steps,
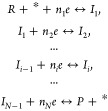
1where *R* and *P* denote the reactant and product, * denotes free sites on the catalyst surface for adsorption, *I*_*i*_ is an adsorbed intermediate with coverage θ_*i*_, and *n*_*i*_ is the number of electrons transferred in *i*th step.

The net rates of elementary steps are given by
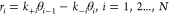
2where θ_0_=θ_*N*_ denotes the coverage of free sites. Rate constants, *k*_+*i*_ and *k*_–*i*_, are calculated based on transition state theory,
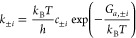
3Here, *c*_±*i*_ represents an assembled concentration factor for all species involved in the forward and backward reactions other than the vacancies, adsorbates, and electrons.

Activation barriers, *G*_*a,i*_, can be written using the Brønsted–Evans–Polanyi (BEP) relation,^31,50^
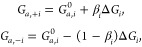
4where *G*_*a,i*_^0^ is the activation energy of step *i* under standard conditions (1 bar pressure, pH = 0) for chemical steps, and at equilibrium potential under the standard conditions for electrochemical steps, β_*i*_ the transfer coefficient, and Δ*G*_*i*_ the reaction Gibbs free energy. For the electrochemical steps, Δ*G*_*i*_ shifts with potential in the following way,
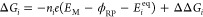
5with *E*_M_ being the applied potential relative to the standard hydrogen electrode (SHE), and ϕ_RP_ the electric potential at the RP. *E*_*i*_^eq^ is the equilibrium potential of step *i* and is calculated using the Nernst equation,
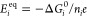
6with Δ*G*_*i*_^0^ being the reaction free energy of step *i* under the standard conditions, which can be obtained from first-principles calculations and thermodynamic modeling, as implemented in the computational hydrogen electrode scheme of Nørskov and coelleagues.^7,31^ Additionally, recent studies underscore the significance of lateral interactions,^51^ electric field,^52^ or electrode surface charge in influencing the Gibbs free energies of adsorbates.^19^ These effects can be incorporated into this framework by introducing the term ΔΔ*G*_*i*_, which is a function of coverages, surface charge density or electric field.

Under steady-state conditions, we have
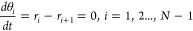
7

Combined with the conservation of adsorption sites, *i.e*., ∑_i = 1_^N^ θ_*i*_ = 1, eq 7 can be solved to obtain θ_*i*_ and *r*_*i*_. It is worth noting that an analytical solution can be derived for reactions with first-order kinetics. Further manipulation of the analytical solution leads to the concept of rate-determining-term (RDT).^1^ The steady-state current density is written as
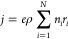
8with ρ being the number density of active sites at the electrode surface.

Several variables of the microkinetic model, including *c*_±*i*_, ϕ_RP_, surface charge density and electric field, need to be determined with the LRE model. The modified Nernst–Planck equation, which takes into account steric effects, can be employed to model the mass transport of species,^53^
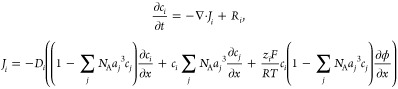
9where *R*_*i*_ is the source term due to homogeneous reactions (*e.g*., conversion between CO_2_ and HCO_3_^*–*^ in CO_2_RR), *J*_*i*_ the flux of species *i*, *D*_*i*_ the diffusion coefficient, *a*_*j*_ the effective diameter, *z*_*i*_ the charge number, and ϕ the electric potential. The Nernst–Planck equation is complemented by the Poisson equation,
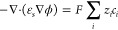
10with ε_*s*_ being the permittivity of electrolyte. A more comprehensive treatment of a modified Poisson–Nernst–Planck framework that takes into account solvent polarization effects can be found in ref (53).

As shown in Figure 2, the model domain spans between the reaction plane and the solution bulk, with the diffusion layer thickness determined according to experimental conditions.^54^ To solve the Poisson–Nernst–Planck (PNP) equations, boundary conditions are needed. The right boundary is situated in the solution bulk, where concentrations match bulk concentrations, and the electric potential is taken as the reference potential, namely, ϕ = 0. The left boundary is located at the RP, and the fluxes at this side correlate with the current densities obtained from the microkinetic model,
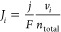
11where *v*_*i*_ is the respective stoichiometric number of species *i*, with *v*_*i*_ being negative for reactants and positive for products, and *n*_total_ is the total number of electrons involved in the reaction.

The EDL structure is incorporated into the boundary condition for the electric potential,
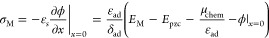
12with σ_M_ being the free surface charge density, *E*_pzc_ the potential of zero charge (pzc), ε_ad_ the permittivity of the adlayer, and δ_ad_ the thickness of the adlayer. It has been pointed out by Johnson et al. that the surface charge boundary conditions are often misused in L4 models, leading to incorrect conclusions regarding EDL effects.^48^ The inconsistencies arise from the adoption of inaccurate permittivity for the adlayer or the artificial imposition of an electric potential at the RP. In our works, we estimate the key parameters, *i.e*., ε_ad_ and δ_ad_, based on ab initio molecular dynamics (AIMD) simulations. In addition, to the best of our knowledge, our approach is unique in that it considers the surface dipole moment induced by partially charged chemisorbates, μ_chem_. It has been shown that μ_chem_ markedly modifies the surface charging relation and the LRE.^55^ Furthermore, it is worth noting that a more detailed EDL structure that accounts for the first water layer can also be integrated into eq 12.^55^

The overall model parameters can be categorized into three groups. The first group of parameters describe reaction properties, including adsorption energies, activation barriers, transfer coefficients and lateral interaction coefficients between adsorbates. These parameters are derived primarily from DFT calculations. The second group characterizes the EDL structure, encompassing the permittivity and thickness of adlayer, effective diameters of solvated ions, and dipole moments of adsorbates. These parameters can be obtained with the aid of DFT and AIMD. The third group defines the mass transport characteristics, incorporating diffusion coefficients, bulk concentrations, and diffusion layer thickness. These parameters relate to experimental conditions. For instance, diffusion layer thicknesses depend on the rotation speed for the rotating disk electrode (RDE) systems.

With the provided inputs, the overall model can be solved self-consistently, *e.g*., using COMSOL Multiphysics. The comprehensive array of model outputs includes partial current densities, adsorbate coverages, surface charging relation, potential distribution, concentration and pH profiles, and more. Furthermore, the framework is suitable for investigations of selectivity aspects, as any number of competing reactions can be included in the microkinetic model, although this aspect lies beyond the scope of this Account.

## Insights into Electrode Kinetics

The hierarchical framework has been applied to several electrocatalytic reactions, *e.g*., ORR, CO_2_RR, OER, FAOR and HPRR in recent years. In the following sections, we illustrate how our approach helps understand various kinetic phenomena, including potential-dependent Tafel slopes, cation effects and pH effects.

## Potential-Dependent Tafel Slopes

Potential-dependent Tafel slopes are prevalent across many reactions,^13^ constituting a topic of unattenuated discussions in electrocatalysis. The conventional view relates the potential-dependent Tafel slopes to transitions of the RDS. For a sequence of consecutive elementary steps, the Tafel slope *b* is related to the “overall transfer coefficient” α,^56^
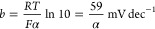
13at room temperature. Here, α = *n*_*f*_+β_*r*_*n*_*r*_, with *n*_*f*_ being the number of electrons released before the RDS, *n*_*r*_ the number of electrons involved in the RDS, and β_*r*_ the transfer coefficient of the RDS.

Provided with an *a priori* reaction mechanism, eq 13 allows identifying the RDS from the Tafel slope. For instance, a Tafel slope of ∼118 mV/dec is usually taken as evidence for the first electron transfer as the RDS, a Tafel slope of ∼59 mV/dec the second chemical step following an electrochemical step as the RDS, and a Tafel slope of ∼39 mV/dec the second electron transfer step as the RDS. It is important to note that this designation assumes β_r_ = 0.5, which has no fundamental justification; furthermore, Marcus theory of electron transfer shows that  (with η being overpotential and λ being solvent reorganization energy), which changes with overpotential.^57^

Additionally, the above view relies on two assumptions. First, it presupposes a slow step that controls the net rate of the reaction, and all other steps are in quasi-equilibrium. Second, the coverage of adsorbates on the catalyst surface is assumed to be negligible.^13,14^ In some cases, the second assumption is alleviated by determining adsorbate coverages under quasi-equilibrium conditions.^13^ However, quasi-equilibrium conditions implied in both assumptions contradict the fact that the reaction has a net rate and that all elementary steps proceed with the same rate.^30^ To overcome these problematic assumptions, the development of a microkinetic model, which considers the thermodynamics and kinetics of all elementary steps, becomes compelling.^1,30^

**Figure 3:**
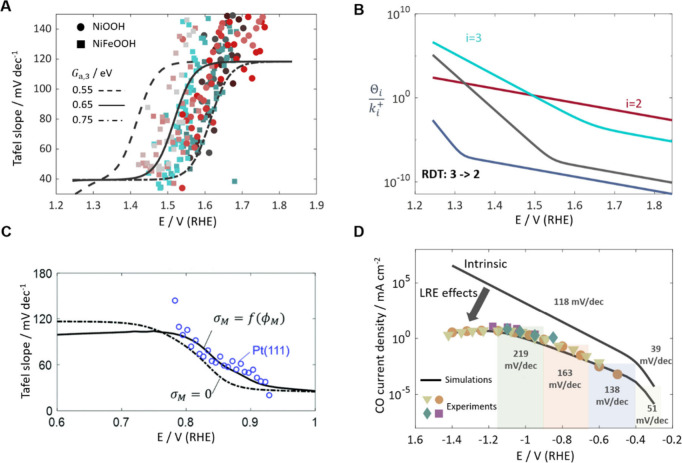
(A) Tafel slopes of the OER. The three lines represent intrinsic Tafel slopes derived from the microkinetic model with different kinetic parameters. The symbols denote experimental data measured on NiOOH and NiFeOOH catalysts. (B) The resistance terms, , of the OER. (C) Tafel slopes of the ORR. The dotted line represents intrinsic Tafel slopes. The solid line represents apparent Tafel slopes that account for the EDL effects. The symbols are experimental data measured on Pt(111). (D) Comparison of simulations (solid lines) and experiments (symbols) for the CO partial current density of CO_2_RR at Ag. The intrinsic Tafel slope is 39 mV/dec at low overpotential and 118 mV/dec at high overpotential. The Tafel slopes with the LRE effects at different potential ranges are annotated. Panels (A) and (B) are adapted with permission from ref (1). Copyright 2021 Elsevier. Panel (C) is reproduced with permission from ref (50). Copyright 2018 Royal Society of Chemistry. Panel (D) is adapted with permission from ref (3). Copyright 2021 American Chemical Society.

The presented hierarchical framework has been demonstrated to be capable of deciphering the potential-dependent Tafel slopes of the OER (Figure 3A), ORR (Figure 3C) and CO_2_RR (Figure 3D). The common trend observed is that the Tafel slope increases with overpotential. This can be rationalized through the RDT analysis of intrinsic multistep kinetics.^1^

For the specific example of the OER, the inverse reaction rate, *i.e*., the reaction resistance, is given by
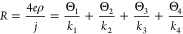
14with the thermodynamic factors
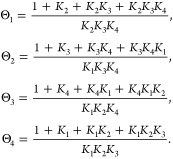
15Here, *K*_*i*_=*k*_*i*_/*k*_*-i*_ are equilibrium constants. eq 14 decomposes the overall reaction resistance into four resistive terms. These terms usually differ by several orders of magnitude, as illustrated in Figure 3B, with the largest term determining the overall reaction resistance and thus the net rate. This term is defined as the RDT. The RDT of the OER is shown to change with potential, with a switch from *i* = 3 to *i* = 2 at 1.5 V (Figure 3B). This transition constitutes the fundamental origin of potential-dependent Tafel slopes.^1^ Specifically, in the low overpotential region (1.23–1.50 V),  is the RDT. Detailed parametrization reveals that the dominant term in the numerator of Θ_3_ is *K*_4_*K*_1_. Therefore,  simplifies to , which results in α = 1.5 and *b* = 39 mV dec^–1^. In the high overpotential region (above 1.50 V),  becomes the RDT, with the dominant term in the numerator of Θ_2_ being *K*_3_*K*_4_*K*_1_. Therefore,  simplifies to , resulting in α = 0.5 and *b* = 118 mV dec^–1^.

In addition to the intrinsic multistep kinetics, the LRE also impacts measured Tafel slopes. Moreover, the influence of the LRE is unavoidable due to the presence of the EDL. We depict the LRE effects on the Tafel slopes of the ORR in Figure 3C and of the CO_2_RR in Figure 3D. Mass transport effects tend to increase the Tafel slope with increasing current density, especially for reactions with low reactant concentration in bulk solution (*e.g*., CO_2_RR), due to the concentration decrease at surface. At low overpotentials, the EDL effects are usually predominant. For instance, the intrinsic Tafel slope is 39 mV/dec for the ORR at 0.9 V and the CO_2_RR at −0.3 V, while the apparent Tafel slope is close to 59 mV/dec due to the EDL effects. However, 39 mV/dec and 59 mV/dec imply different RDSs in the conventional analysis. Furthermore, lateral interactions from competitive adsorbates^3,51^ and surface charging effects on adsorption energies^18^ are revealed as significant influences on the Tafel slope.

Combined, we conclude that the apparent Tafel slope is a composite reaction parameter and may be a poor activity metric as it is influenced by several interacting factors, including the thermodynamics and kinetics of multiple elementary steps, and the LRE effects. The proposed framework aids in revealing the mechanisms behind the apparent potential-dependent Tafel slopes, remedying an oversimplified analysis of the RDS from the Tafel slope.

## Cation Overcrowding Effect

The effects of cation type and concentration have been explored for various electrocatalytic reactions.^33,34,58−60^ To elucidate the observed cation effects, several mechanisms have been proposed. For instance, Singh et al. attributed cation effects in the CO_2_RR to cation hydrolysis. Specifically, cations with a smaller hydrated size can buffer the interfacial pH near cathode more effectively.^34^ Using the modified Poisson–Boltzmann model, Ringe et al. illustrated that the electrode surface charge density is more negative for Cs^+^ than Li^+^, which, in turn, enhances the stability of intermediates, *e*.*g*., *CO_2_ and *COOH, in CO_2_RR.^19^ Similar surface charge effects can also explain cation effects in the HER,^33^ ORR^58^ and OER.^59^ Huang et al. rationalized cation-dependent kinetics of the HER/HOR by considering the influence of cations on the interfacial water structure and H-bonding network.^44^ Furthermore, Qin et al. proposed that the CO_2_RR proceeds through an inner-sphere electron transfer pathway in the presence of alkali cations and, in contrast, through an outer-sphere electron transfer pathway in systems without cations.^60^

Most of the above mechanisms assume that the binding energies of adsorbed intermediates are affected by the electric field, which is then modulated by the cations. Following this line of thermodynamic binding-energy approach, we would expect opposite sequences of cation size effects for metals on the left and right legs of the volcano plot. Specifically, for metals on the right leg of the volcano plot, the activity follows the sequence of Li^+^ < Na^+^ < K^+^ < Cs^+^. Conversely, for metals on the left leg of the volcano plot, the activity should follow the sequence of Li^+^ > Na^+^ > K^+^ > Cs^+^. Xue et al. observed opposite trends of cation size effects on the HER at Pt or Au, which adsorb hydrogen too strongly or too weakly, respectively.^33^ However, opposite trends are absent for the CO_2_RR. In particular, for CO_2_RR to HCOOH, Sn locates at the peak of the volcano plot.^61^ Therefore, enhancing the adsorption of the key intermediate, *OCHO, would be expected to decrease the activity. In contrast, since Ag lies at the right leg of the volcano plot, enhancing the adsorption of *OCHO would increase its activity. However, experiments have shown that the CO_2_RR to HCOOH follows the sequence of Li^+^ < Na^+^ < K^+^< Cs^+^ at both Sn and Ag.^32^ This discrepancy has motivated us to look beyond the binding-energy approach and introduce an electrostatic factor, *i*.*e*., the cation overcrowding effect, into consideration. This mechanism was previously acknowledged by Frumkin et al. in the study of peroxydisulfate anion reduction when the surface charge is very negative.^45^ We demonstrated that the cation overcrowding effect offers an alternative or at least complementary explanation to previously observed cation effects.

**Figure 4:**
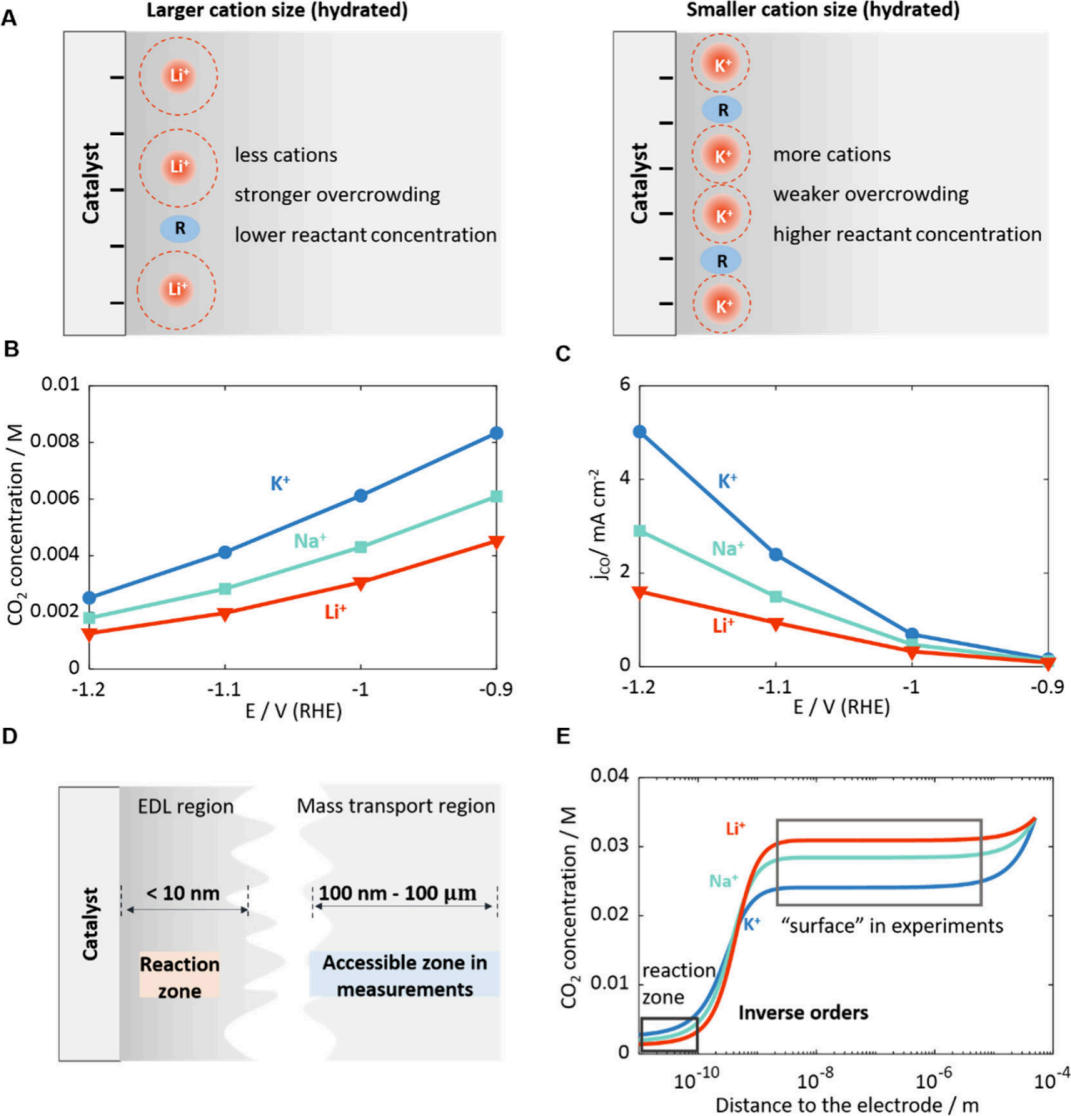
(A) Schematic illustration of the cation overcrowding effect in electrocatalysis. Cations accumulated near the negatively charged surface diminish the space for reactants, resulting in a decrease in the reactant concentration. Moreover, this effect is more pronounced for cations with a larger hydrated size. (B–C) Cation effects on CO_2_RR at Ag in 0.1 M KHCO_3_/NaHCO_3_/LiHCO_3_ solutions: (B) CO_2_ concentration at the reaction plane; (C) model-derived CO current density. (D) Schematic illustration of the difference between the most probable reaction zone and the accessible zone in experimental measurements. (E) Distribution of CO_2_ concentration in solutions at −1.2 V versus reversible hydrogen electrode (RHE). Panels B, C and E are adapted with permission from ref (3). Copyright 2021 American Chemical Society.

The overcrowding effect describes how cations accumulating exceedingly near the negatively charged surface diminish the space for reactants and influence the local electrostatic potential and electric field.^2^ Specifically, the free space for species other than cations is (1-*N*_*A*_*a*_*c*_^3^*c*_*c*_), with *a*_*c*_ being the effective diameter and *c*_*c*_ the concentration of cations. Theory and simulations accounting for the cation size have shown that this effect is more pronounced for cations with a larger hydrated size,^2^ as depicted in Figure 4A. Consequently, the concentration of reactant, *e*.*g*., CO_2_ for CO_2_RR and OH^–^ for OER, at the surface follows the order Li^+^ < Na^+^ < K^+^ (Figure 4B), which results in the same order of activity. Despite its simplicity, this rationale was shown to be relevant in explaining the cation effects in the CO_2_RR at Ag (Figure 4C),^3^ and the OER at Ni-based catalysts.^2^

Various experimental techniques have been employed to detect the local reactant concentration or the local pH, such as surface enhanced infrared absorption spectroscopy, Raman spectroscopy, scanning electrochemical microscopy.^24−26^ However, it is essential to acknowledge that in these experiments, the term “surface” typically refers to somewhere within the diffusion layer. The distance of the probe position to the catalyst surface varies from hundreds of nanometers to hundreds of micrometers,^24^ which is still far out from the reaction zone within the EDL. This discrepancy in the designation of the “surface” concentration, as depicted in Figure 4D, may lead to confusion. For instance, Malkani et al. observed that the “surface” CO_2_ concentration follows the sequence of Li^+^ > Na^+^ > K^+^. At first glance, this shows the opposite trend to our simulations in Figure 4B. This superficial inconsistency can be resolved by distinguishing the “surface” concentration in experiments and in simulations. As shown in Figure 4E, our simulations show that the CO_2_ concentration in the diffusion layer, which corresponds to the “surface” in experiments, follows the order of Li^+^ > Na^+^ > K^+^. The concentration in this diffusion region is determined by mass transport effects; the above concentration sequence is a direct consequence of the fact that the current density of the CO_2_RR follows the sequence Li^+^ < Na^+^ < K^+^. However, electrostatic interactions and the cation overcrowding effect dominate within the EDL, leading to the inverse order of CO_2_ concentration.

## pH Effects

The influence of solution pH on electrocatalytic reactions is multifaceted, including intrinsic pH effects and local pH effects. In broad terms, changes in solution pH impact reaction kinetics by inducing shifts in both proton activity and absolute potential of the electrode (*i.e*., versus the SHE). Given that many electrocatalytic reactions involve proton and electron transfers, variations in pH play a pivotal role. Moreover, the proton donor or oxidant involved in the reaction may transition from proton/hydroxyl to water molecules when pH varies in a wide range. Additionally, for reactants engaged in acid-alkaline equilibrium, such as formic acid and formate, the concentration of the reactant is influenced by the solution pH. These influences are termed as intrinsic pH effects as they collectively shape the overall properties of electrocatalytic reactions. These intrinsic effects have been widely employed to understand pH effects in various electrocatalytic reactions, including CO_2_RR,^17^ electrochemical carbon monoxide reduction,^62^ HER,^37^ ORR,^63^ OER,^36^ and FAOR.^35,64^

In addition to these intrinsic pH effects, we have emphasized the importance of considering local pH effects, namely, the pH effects arising in the LRE.^2,4,49^ On one hand, the local pH shifts with the reaction rate due to the production/consumption of protons, and this pH shift is more pronounced in the intermediate pH range than in strongly acidic or alkaline contions.^65^ On the other hand, the pzc on the reversible hydrogen electrode (RHE) scale increases with pH,
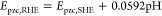
16Here, *E*_pzc, SHE_ is the pzc on the SHE scale. Consequently, the surface charge is more negative at higher pH at the same potential versus RHE, resulting in the change of EDL properties.

Thermodynamic equilibrium conditions predict that the OER activity should be independent of pH on the RHE scale since it is a proton-coupled electron transfer (PCET) reaction.^36^ However, experiments show that the activity increases with pH.^36^ This discrepancy can be understood by considering the EDL effects.

**Figure 5:**
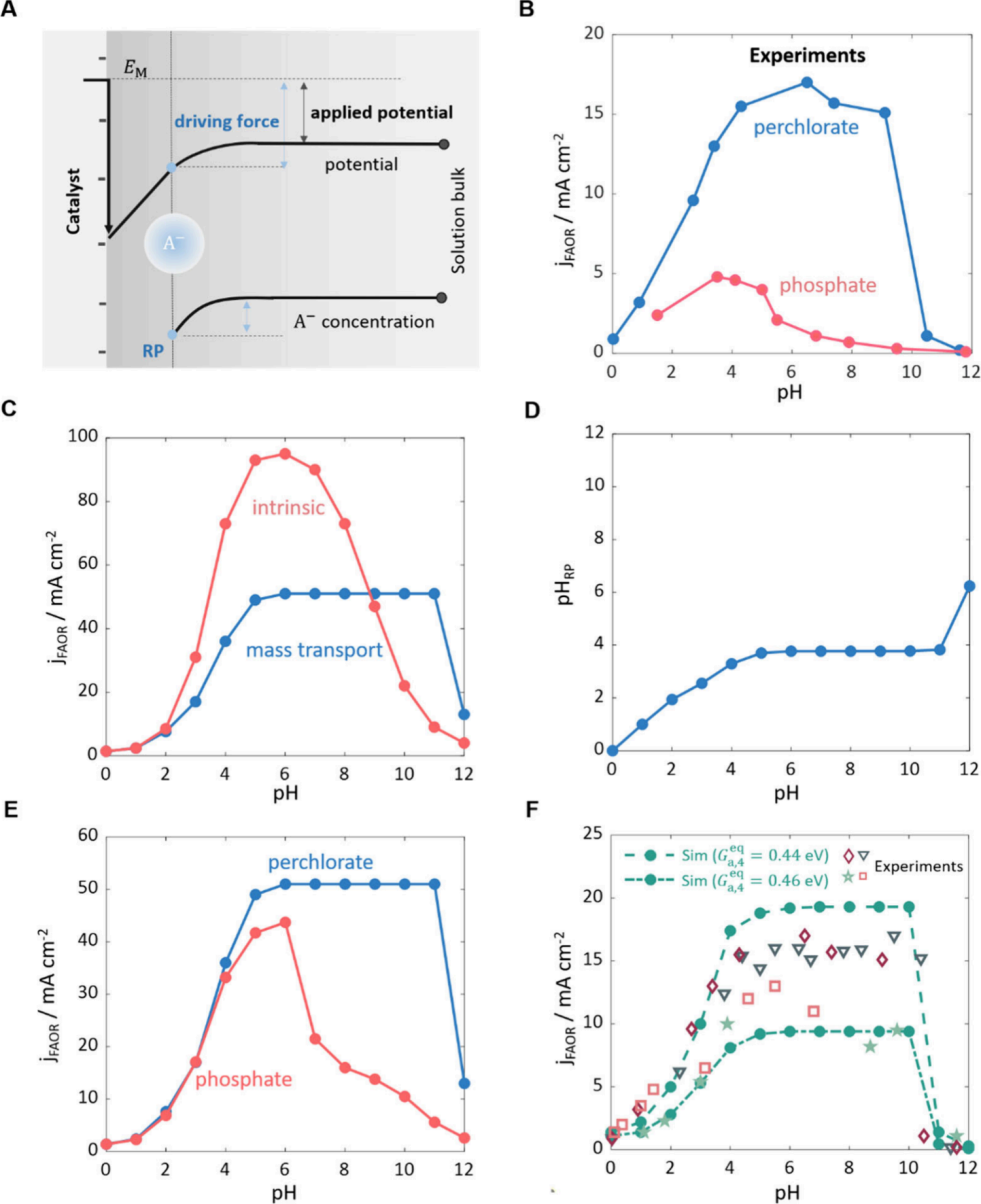
(A) Schematic of the Frumkin theory for electrocatalytic reactions. (B–F) pH effects on the FAOR at Pt electrode: (B) pH-activity relations of the FAOR observed in experiments, which are bell-shaped in phosphate solution and trapezoidal-shaped in perchlorate solution;^35^ (C) intrinsic activity-pH relation without considering LRE effects, and activity-pH relation accounting for mass transport effects in perchlorate solution; (D) comparison of the pH in bulk solution and the pH at the RP; (E) model-derived activity-pH relations in perchlorate solution and phosphate solution accounting for mass transport effects; (F) comparison between the experiments and the simulations with the full-level model in perchlorate solutions. Panels C–F are adapted with permission from ref (4). Copyright 2023 American Chemical Society.

For the electrochemical oxidation of anions, such as OH^–^ in the OER, the negative surface charge induces two competing effects, as per Frumkin effects (Figure 5A): it increases the driving force and decreases the surface concentration of anions (opposite for positive surface charge). The outcome of these competing effects determines the promotion or inhibition of activity. Furthermore, Frumkin effects depend on the pH, as it modulates the surface charge, *i.e*., the surface charge is more negative at higher pH. For the case of the OER at NiOOH, the effect of increasing the driving force is more pronounced. As a result, the activity exhibits an increase as the surface charge becomes more negative, and thus at higher pH.^2^

However, Frumkin effects have been revealed as being insignificant in the case of FAOR, as the competing effects more or less cancel each other out. Instead, the mass transport-induced local pH shift emerges as a crucial factor that influences observed pH effects.^4^ Although the FAOR has been studied for many decades as a model reaction, the relation between its activity and pH remains controversial. Joo et al. first reported that the activity-pH relation of FAOR at Pt exhibits a bell shape with the peak at the p*K*_a_ of formic acid (∼4).^64^ Their study considered phosphate solutions with pH ranging from 0 to 12. It was explained that the activity increases with pH when pH < p*K*_a_ since the concentration of HCOO^–^ increases, which is the main reactant. The site-blocking effect of OH adsorption becomes significant when pH > p*K*_a_; therefore, the activity decreases with pH in this range. However, a trapezoidal-shaped activity-pH relation with a plateau between pH = 5 and 10 was observed when perchlorate solution was used (Figure 5B).^35^ This observation challenged the previously proposed mechanism.

The complexity of pH effects in this model system arises from multiple interacting factors, including pH-dependent thermodynamics and reaction kinetics of multiple steps, and pH-dependent LRE effects. This situation has motivated us to build a hierarchical model in an incremental manner allowing different factors that control the overall pH effect to be disentangled. Our analysis began with exclusive consideration of the microkinetics in perchlorate solutions, in which the specific adsorption of electrolyte anions can be avoided. Based on this L2 modeling, we obtained the intrinsic activity-pH relation without accounting for the LRE. As shown in Figure 5C, the intrinsic activity-pH relation is bell-shaped with a peak at pH = 6, which is inconsistent with the observed trapezoidal shape. We then added mass transport effects to the model, namely, modeling on the L3. Figure 5D indicates that the pH at the reaction plane (pH_RP_) is much lower than the pH in the bulk solution (pH_bulk_), as the FAOR generates protons. Moreover, the pH_RP_ remains almost constant at pH_RP_ = 4 in the range of 5 < pH_bulk_ < 11. This local pH shift induces a transformation of the activity-pH relation from bell shape to trapezoidal shape, yielding qualitative agreement with the experimental trend (Figure 5C).^35^ For the activity-pH relation in phosphate solution, there are two additional electrolyte effects, *i.e*., the buffering effect and the specific adsorption of phosphate anions. By incorporating both effects, we captured the experimental trends in phosphate solution with the activity being lower than that in perchlorate solution and the activity-pH relation being bell-shaped (Figure 5E). Furthermore, the site-blocking effect of the specific adsorption of phosphate anions was revealed to be the determining factor.

However, we noticed that the simulated activity is approximately three times higher than the experimental data, which cannot be explained by Frumkin effects. Therefore, we suggested that specific EDL effects beyond Frumkin corrections are likely responsible for this. By incorporating the surface charging effect on adsorption energies of formate, the model captures the experiments quantitatively in both perchlorate solution (Figure 5F) and phosphate solution.^4^

For the H_2_O_2_ redox reaction, pH effects were shown to arise from the pH-dependent surface charging effects, which were studied using AIMD at electrified Pt(111)-water interfaces.^49^ The negative and positive surface charge conditions were simulated by introducing a lithium ion and a fluorine ion in the water layer, respectively. It was revealed that the negative surface charge repels the O–O bond of H_2_O_2_ farther away from the electrode surface, leading to a higher activation barrier for breaking the O–O bond. When the applied potential shifts negatively, the driving force of HPRR increases, which leads to the decrease of the activation barrier and thus promotes the activity. Concurrently, the surface charge becomes more negative, increasing the activation barrier and suppressing the reaction. These two competing effects cause the nonmonotonic (first increasing and then decreasing) activity of HPRR with the negative shift of electrode potential. The activity suppression effect caused by negative surface charge is also responsible for the pH effects of HPRR. As the surface charge becomes more negative with increasing pH, the onset of the suppression effect shifts to more positive potential for higher pH. Consequently, the activity decreases at more positive potential at higher pH, which is consistent with experimental observations.^66^

Given the above analysis, we underscore the importance of considering the variation of LRE when the solution pH changes. Fluctuations in local pH and surface charging relation induced by pH changes could be the determining factors of apparent pH effects.

## Summary and Outlook

The multiple interrelated factors discerned in this Account, including thermodynamics, multistep kinetics, mass transport, and EDL charging, determine kinetic phenomena in electrocatalytic reactions, such as potential-dependent Tafel slopes, cation effects, and pH effects. We have presented a hierarchical framework that integrates two essential modules: a microkinetic model that incorporates the thermodynamics and kinetics of all elementary steps and a LRE model that accounts for the microscopic EDL structure and macroscopic mass transport in a unified manner. So far, applications of this framework to various electrocatalytic reactions have yielded vital insights into potential-dependent Tafel slopes, cation effects, and pH effects. From our perspective, it is crucial to start from a holistic, unbiased view when deciphering the physical origins behind various reaction phenomena.

Until now, our framework has been applied to planar electrodes with static structure and operated in the steady state. Several important extensions to the framework should be made in the stride toward realism with realistic structure and dynamic conditions.^67^ First, time-dependent methods, *e.g*., pulsed electrolysis, have been acknowledged to be effective in improving the activity and selectivity of CO_2_RR^68^ and ORR.^69^ Second, the catalyst has been revealed to dynamically reconstruct, instead of being static, during reactions.^70^ Third, electrochemical energy conversion technologies typically employ supported nanoparticle catalysts, requiring a proper treatment of synergistic effects due to the overlap of the EDLs from catalytic nanoparticles and support material.^71^ Fourth, the catalyst in gas diffusion electrodes is not simply embedded into an aqueous electrolyte (as usually considered in model studies) but it is surrounded by a thin ionomer film that creates a water-filled nanogap region around the catalyst.^72^ This interfacial configuration is crucial for understanding LRE effects at catalyst-ionomer interfaces. Future endeavors in addressing these complexities should take advantage of recent developments in theory and modeling of electrochemical phenomena. For instance, the thermodynamics and kinetics of elementary steps involved in the microkinetic model can be calculated with increasing accuracy using grand-canonical DFT.^73^ In addition, Marcus–Hush-Chidsey theory should be employed instead of the Butler–Volmer equation to describe the electron-transfer kinetics at high overpotentials^57^ and in the case of the solvent reorganization energy changing markedly with electrode potential.^21^ Furthermore, the mean field EDL model can be refined and complemented by incorporating atomistic and molecular details obtained from first-principles calculations.^16^ Finally, development of high-performance computation infrastructure and rapidly emerging machine learning techniques pave the way toward handling complexities of real-world electrocatalytic systems.^74^
